# Boolean network sketches: a unifying framework for logical model inference

**DOI:** 10.1093/bioinformatics/btad158

**Published:** 2023-04-02

**Authors:** Nikola Beneš, Luboš Brim, Ondřej Huvar, Samuel Pastva, David Šafránek

**Affiliations:** Faculty of Informatics, Masaryk University, Brno 602 00, Czech Republic; Faculty of Informatics, Masaryk University, Brno 602 00, Czech Republic; Faculty of Informatics, Masaryk University, Brno 602 00, Czech Republic; Institute of Science and Technology Austria, Klosterneuburg 3400, Austria; Faculty of Informatics, Masaryk University, Brno 602 00, Czech Republic

## Abstract

**Motivation:**

The problem of model inference is of fundamental importance to systems biology. Logical models (e.g. Boolean networks; BNs) represent a computationally attractive approach capable of handling large biological networks. The models are typically inferred from experimental data. However, even with a substantial amount of experimental data supported by some prior knowledge, existing inference methods often focus on a small sample of admissible candidate models only.

**Results:**

We propose Boolean network sketches as a new formal instrument for the inference of Boolean networks. A sketch integrates (typically partial) knowledge about the network’s topology and the update logic (obtained through, e.g. a biological knowledge base or a literature search), as well as further assumptions about the properties of the network’s transitions (e.g. the form of its attractor landscape), and additional restrictions on the model dynamics given by the measured experimental data. Our new BNs inference algorithm starts with an ‘initial’ sketch, which is extended by adding restrictions representing experimental data to a ‘data-informed’ sketch and subsequently computes all BNs consistent with the data-informed sketch. Our algorithm is based on a symbolic representation and coloured model-checking. Our approach is unique in its ability to cover a broad spectrum of knowledge and efficiently produce a compact representation of all inferred BNs. We evaluate the method on a non-trivial collection of real-world and simulated data.

**Availability and implementation:**

All software and data are freely available as a reproducible artefact at https://doi.org/10.5281/zenodo.7688740.

## 1 Introduction

Boolean networks (BNs) (Kauffman 1969, Thomas 1973) represent a simple yet expressive formalism for modelling various processes in living cells. This is why BNs are gaining significant interest in the scientific community, especially in the area of computational systems biology (e.g. Grieb et al. 2015). Each BN consists of Boolean variables with associated Boolean update functions governing their behaviour. The goal of BN inference (also termed synthesis) is to reconstruct the network from experimental observations and other prior knowledge. BN inference is a crucial subject with regard to any practical application of BNs.

As termed by Gunawardena (2014), we can generally recognize two dominant modelling strategies: ‘forward’ and ‘reverse’ modelling. Reverse modelling starts from experimental data and seeks to identify the causalities in this data using a mathematical model. According to Gunawardena (2014), reverse modelling often suggests new molecular components or interactions but typically cannot identify conceptually entirely new ideas. Meanwhile, forward modelling, also known as literature-based modelling, starts from a set of known or suspected causalities and seeks to obtain a predictive model based on these assumptions. This makes forward modelling capable of formulating new abstractions for understanding high-level system behaviour, e.g. homeostasis, feedback, or canalization.

We believe neither approach is sufficient for reliable inference of large-scale BNs. The often unpredictable long-term impact of combining multiple assumptions makes scaling the forward modelling approach to real-world cases hard and error-prone. Nevertheless, the availability of such assumptions is a valuable resource that must not be neglected (Peng et al. 2010). At the same time, inference from experimental data is limited by the number of measurements (Cheng and Zhao 2011, Cheng et al. 2011) and their quality (Huang et al. 2022). Despite the recent progress in technologies allowing observation of gene expression, it is still not easy nor cheap to obtain the necessary data in sufficient volume and precision. In addition, the experimental data are typically short and noisy (Bar-Joseph 2004). More relevant observations are, however, available when the underlying network is at a steady state, e.g. see gene expression profiles of melanoma (Bittner et al. 2000). Such states typically correspond to network attractors: parts of the state space that cannot be escaped. We thus primarily assume, though not exclusively, that the experimental data represent steady-state data.

In this article, we advocate a combination of forward and reverse modelling (Peng et al. 2010). The general idea is to unify the literature-based knowledge and the knowledge gained from the experimental data. To that end, we propose Boolean network sketches as a new formal instrument for the inference of BNs. A network sketch integrates partial knowledge about the network’s topology and the update logic (obtained through, e.g. a biological knowledge base or a literature search), as well as dynamical restrictions representing knowledge or assumptions about the properties of the network’s transitions (e.g. attractor landscape), and restrictions on the model dynamics given by the measured experimental data. A unique feature of our approach is that the modeller can also explicitly formulate, as a part of prior knowledge, what is ‘not known’. Our new inference method starts with an ‘initial’ sketch that corresponds to the prior literature-based knowledge only. Subsequently, it is extended by adding restrictions representing experimental data resulting in the ‘data-informed’ sketch. The inference procedure then identifies BNs that are consistent with the data-informed sketch.

We assume that the experimental data that enter the inference procedure are already binarized (Shmulevich and Zhang 2002), and the measurements represent either the system’s steady states or time-series experiments. We consider asynchronous BNs since they are more biologically appropriate (Saadatpour et al. 2010). In asynchronous dynamics, attractors can be classified as ‘stable’ (all variables are fixed), ‘cyclic’ (values of some variables oscillate), and ‘complex’ (values of some variables behave unpredictably). However, note that in a typical experiment, not all system variables are measurable. Thus, an apparent steady state may correspond to a cyclic or complex attractor when the unstable variables are not observed. Therefore, even if we only consider steady-state data, it is still necessary to consider all network attractors, not just the stable states.

We would also like to stress that the notion of a BN sketch is not strictly tied to the asynchronous semantics. After a simple modification of the core engine, our method is adaptable to any semantics that produces a state-transition graph [e.g. synchronous, generalized asynchronous—Chatain et al. (2018a), or most permissive—Chatain et al. (2018b), and their respective sub-variants].

The contribution of our article is two fold: First, we formally introduce BN sketches as a means of rigorously combining partial knowledge about the Boolean model with measurement data. Second, we formulate a symbolic binary decision diagram (BDD)-based procedure utilizing coloured model checking (Brim et al. 2015) and attractor detection algorithms (tool AEON) (Beneš et al. 2020) that computes all BNs consistent with a given network sketch. The BDD representation allows us to compute all these networks at once. The algorithm’s result is an ensemble of all the candidate logical models that is amenable for further processing. Such processing may involve, e.g. picking a particular network consistent with all the desired requirements, designing additional experiments to further reduce the candidate set or discovering common properties shared by all the candidate networks.

*Related work*. In the domain of continuous models, the interplay of forward and reverse modelling has been studied, e.g. in Peng et al. (2010), albeit in a very application-specific manner. In Ostrowski et al. (2016) and later in Chevalier et al. (2019), the authors consider a similar exhaustive BN inference problem as this article, arising from an assumed influence graph (IG) and observed reachability properties. To identify the collection of candidate networks, they employ answer-set programming. However, both works focus on more coarse-grained over-approximations of the asynchronous BN semantics. A detailed comparison is available in the supplementary material.

Note that Muñoz et al. (2018) employ specific information on prior knowledge formalized in terms of R-graphs and other structures capturing data. In contrast, our approach is significantly more general and includes universal specifications of many aspects, e.g. dynamical properties.

The closest related work on the inference of logical models with the help of model-checking methods is the framework of abstract Boolean Networks (ABN) introduced in Yordanov et al. (2016) and implemented in RE: IN by Goldfeder and Kugler (2019). ABNs are associated with experimental constraints (corresponding to a subclass of dynamical restrictions in our framework), which makes them comparable with data-informed sketches (see the supplementary material for details). However, on the computational side, RE: IN employs bounded model checking based on satisfiability-modulo-theories, which limits the experimental constraints to basic reachability. In our approach, network sketches employ a richer logic allowing significantly more expressive specifications: steady-state behaviour (attractors), advanced reachability (e.g. monotonicity in between measurements in a time series), and a combination of both (e.g. basins of attraction). Crucially, the synthesis process of Yordanov et al. (2016) is limited to a pre-defined set of “patterns” for update functions and is, therefore, not truly exhaustive.

In general, a distinguishing feature of our approach, thanks to the underlying BDD representation, is our ability to easily obtain all candidate models. This is not possible with methods using logic-based reasoning described above. The efficiency of BDDs in this application stems from their ability to compress various redundancies within Boolean functions. On average, this compression ratio grows with the number of symbolic variables (Newton and Verna 2019). This allows us to efficiently manipulate extremely large sets of BNs, as long as the networks are sufficiently similar. However, this phenomenon only holds on average: counting all monotonic Boolean functions (Dedekind 1897) is a famous example of a simple problem that cannot be efficiently solved using BDDs, or any other known algorithm for that matter.

Traditional inference algorithms based on optimization emphasize network topology inference (dependencies among variables). This includes techniques based on mutual information [tool REVEAL; Liang et al. (1998), and tool ARACNE; Margolin et al. (2006)] or genetic programming (Mendoza and Bazzan 2011). Asynchronous dynamics is supported in, e.g. Gao et al. (2020) (genetic programming) or Lim et al. (2016) (state-space scoring). However, compared to our approach, these methods only select a single or a small subset of candidate networks without guarantees on the method’s stability or exhaustiveness. Most of the existing inference algorithms target synchronous dynamics, e.g. exhaustive search (Best-Fit) (Lähdesmäki et al. 2003), mutual information (MIBNI) (Barman and Kwon 2017), genetic programming (GABNI) (Barman and Kwon 2018), and AND/OR tree ensembles (ATEN) (Shi et al. 2019) can be considered as well. However, the synchronous case often fails to capture the differences in the time scale of individual updates (Lähdesmäki et al. 2003).

Conceptually, the notion of a BN sketch can also be seen as an enhancement of a prior knowledge network (PKN) (Terfve et al. 2012, Dorier et al. 2016). PKNs summarize known interactions between genes and/or proteins of interest and are usually obtained through literature mining. In Veliz-Cuba et al. (2022), the authors also use the term ‘model prototype’ to refer to incomplete models inferred from time-course data.

## 2 Preliminaries

Our BN inference procedure is based on the notion of a BN sketch. Intuitively, a sketch can be seen as a collection of information a modeller has at his or her disposal when designing a Boolean model. BN sketches can be analysed using various symbolic algorithms to infer the possible exact BNs, called candidate networks.

In the following, we use B to denote the set of Boolean values {0, 1} and $B^{n}$ to denote the set of Boolean-valued vectors of length *n*. Given such a vector $x\in B^{n}$, we write *x_i_* to denote its *i*-th element. Furthermore, x[i↦b] denotes the copy of *x* where the value of the *i*-th element is fixed to *b*. We use the notation $X^{\left(a\right)}$ to denote the arity *a* of *X* (if *X* is a function) or generally the number of variables appearing in some *X* (if *X* is, e.g. a BN). When this number is clear from context, the superscript can be omitted.

### 2.1 Boolean network

A BN $F^{\left(n\right)}$ assumes *n* Boolean variables ${\text{Var}}_{n}=\{v_{1},\ldots ,v_{n}\}$ and consists of *n* Boolean update functions $\left\{F_{1},\ldots ,F_{n}\right\}$ (one for each variable), such that $F_{i}:B^{n}\to B$. Each Boolean-valued vector of $B^{n}$ represents an assignment of Boolean values to the variables of ${\text{Var}}_{n}$. We call the set $B^{n}$ the state space of $F^{\left(n\right)}$, and the members of $B^{n}$ its states.

For each of the functions *F_i_*, we define $\mathrm{dep}(F_{i})\subseteq {\text{Var}}_{n}$ to be the dependency set of *F_i_*. This set contains the network variables that actually influence the output of *F_i_*:


vj∈dep(Fi)⇔∃x∈Bn. Fi(x[j↦1])=Fi(x[j↦0]).

*Asynchronous dynamics* In this article, we focus on the asynchronous updating scheme. This approach assumes that every network variable can be updated independently of the others, as opposed to, e.g. the synchronous updating scheme where all variables are updated together.

Under this assumption, we can construct a directed (asynchronous) state-transition graph $\text{STG}(F^{\left(n\right)})=(S,T)$, where $S=B^{n}$ is the network’s state space, and the transition relation T⊆S×S is defined as follows:


(s,t)∈T⇔s=t∧∃i∈{1,…,n}. t=s[i↦Fi(s)].

Notice that in the resulting graph, every transition “updates” exactly one network variable. For technical reasons, without the loss of generality, we include a self-loop (s,s)∈T whenever $\forall i.\;s_{i}=F_{i}(s)$. We use the standard abbreviations $s\to t,\;s\to^{+}t$ and $s\to^{*}t$ to denote that $(s,t)\in T,\;(s,t)\in T^{+}$ (transitive closure) and $(s,t)\in T^{*}$ (reflexive and transitive closure), respectively. A ‘run’ is an infinite sequence of states *s*_1_, *s*_2_, …such that $\forall i.(s_{i},s_{i+1})\in T$.

*Attractor* The long-term behaviour of a BN is typically captured by the notion of attractor. Formally, an attractor *A* is a bottom (or terminal) strongly connected component of STG(F). In other words, for any two $s,t\in A,\;s\to^{*}t$ (*A* is an SCC), and for all s∈A, s→t implies that t∈A (*A* cannot be escaped). In the asynchronous STG(F), we generally talk about ‘fixed-point’ attractors (*A* is a singleton set), ‘cyclic’ or ‘oscillating’ attractors (*A* is a cycle within STG(F)), and ‘complex’ or ‘disordered’ attractors (*A* is any other set).

## 3 Materials and methods

**Figure 1.**
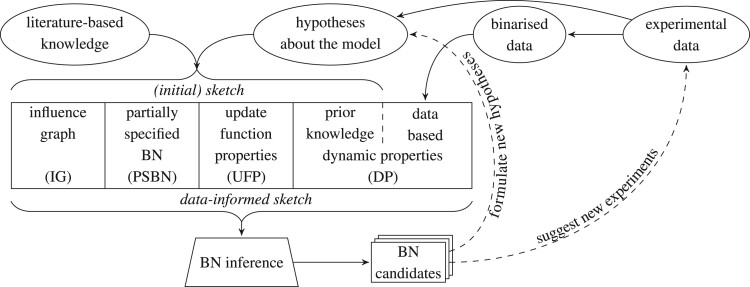
The workflow of our method. The initial sketch is created by combining literature-based knowledge about the model and possible initial hypotheses about the model. The sketch may be further complemented with properties obtained automatically from binarized data, in which case, we also call it the data-informed sketch. Once we run the BN inference procedure, we obtain a set of BN candidates, which we may enumerate and further inspect. This may lead us to the formulation of new hypotheses or suggest new experiments to perform, both of which may further be used to refine the sketch and run the procedure again

The workflow of our method is summarized in Fig. 1. In this section, we explain the workflow. In particular, we first provide a detailed explanation of the individual parts of a BN sketch and then describe the inference procedure that takes such a sketch as an input and provides the set of BN candidates.

### 3.1 BN sketch

**Figure 2.**
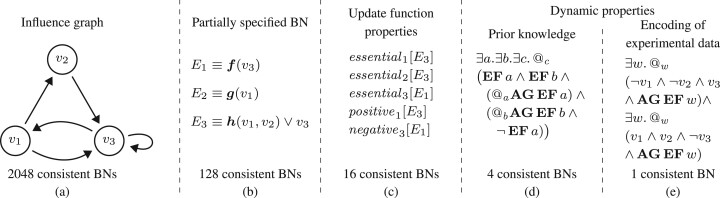
A running example of a BN sketch: (a) an influence graph (3.1.1), (b) a PSBN (3.1.2), (c) UFP (3.1.3), (d) DPs given as HCTL formulae obtained from prior knowledge (3.1.4), and (e) DPs automatically obtained from binarized experimental data (3.1.5). Parts (a)–(d) represent prior knowledge and form the initial sketch; extending the DPs of the initial sketch with (e) gives the data-informed sketch

Now, we formally introduce BN sketches by defining the constituent parts. The core part is made by partially defined BNs. The dynamical restrictions and other assumptions about the system behaviour are described using temporal logic HCTL. To illustrate the concepts, we refer to the running example in Fig. 2.

#### 3.1.1 Influence graph

The high-level structure of the network is given by an influence graph. For a fixed set ${\text{Var}}_{n}$ of *n* Boolean variables, an influence grah (IG) is a binary relation $I^{\left(n\right)}\subseteq {\text{Var}}_{n}\times {\text{Var}}_{n}$. Intuitively, an influence graph specifies the possible dependencies between individual Boolean variables. Given a BN *F*, we say that *F* is consistent with *I* if for every *F_i_* and every $v_{j}\in \mathrm{dep}(F_{i})$, we have that $(v_{j},v_{i})\in I$. The opposite (i.e. $\left(v_{j},v_{i})\in I\Rightarrow v_{j}\in \mathrm{dep}(F_{i}\right)$) is not required, though.

An example of an influence graph is given in Fig. 2a where we fix the set of variables ${\text{Var}}_{3}$. There are 2048 BNs consistent with this graph.

#### 3.1.2 Partially specified Boolean network

Prior knowledge about the model’s behaviour can often be formulated in the form of partially specified update functions. When defining such functions, we may use uninterpreted (fixed but otherwise unknown) function symbols alongside standard Boolean expressions.

Formally, let us assume a set F of function symbols, each with a specified arity. In the following, we use boldface symbols such as $f^{\left(a\right)}$ to denote the members of this set (with the corresponding arity *a*).

A partially specified Boolean network (PSBN) $E^{\left(n\right)}$ again assumes *n* variables ${\text{Var}}_{n}$ and consists of *n* partially specified Boolean expressions $\left\{E_{1},\ldots ,E_{n}\right\}$, where each *E_i_* is defined by the following grammar:


$$E::=0|1|v|\neg E|E\wedge E|f^{\left(a\right)}(E,\ldots ,E).$$

Here, *v* ranges over ${\text{Var}}_{n}$. In practical applications, we also allow standard syntactic abbreviations for other operators like ∨ (disjunction), ⇒ (implication), ⇔ (equivalence), and ⊕ (exclusive or, non-equivalence). Finally, when the arity of ***f*** is 0, this symbol is effectively an unknown constant. As such, we can simply write ***f*** instead of $f^{\left(0\right)}()$.

An interpretation of F is a function I that assigns to each symbol $f^{\left(a\right)}\in F$ a Boolean function $f^{\left(a\right)}$ with the same arity. By fixing a particular interpretation I for a given PSBN $E^{\left(n\right)}$, we substitute a concrete Boolean function for each function symbol in its partially specified Boolean expressions, and thus obtain a standard BN, which we denote by E(I). Finally, we call a BN $F^{\left(n\right)}$ consistent with a PSBN $E^{\left(n\right)}$ if there exists an interpretation I of F such that F=E(I).

An example of a PSBN is given in Fig.2b. Here, we use three function symbols $f^{\left(1\right)},\;g^{\left(1\right)}$, and $h^{\left(2\right)}$. The number of BNs consistent with both the influence graph and the PSBN has now decreased to 128 (this is due to the restriction on the form of *E*_3_).

#### 3.1.3 Update functions properties

Another kind of starting information reflects prior knowledge on properties of Boolean update functions, like monotonicity.

Let *n* be fixed in the following. We use $F^{\left(n\right)}$ to denote the set of all Boolean functions of type $B^{n}\to B$. A Boolean function property is a predicate over $F^{\left(n\right)}$, i.e. a function $F^{\left(n\right)}\to B$. We only work with properties that can be defined in the first-order logic over Booleans. This is sufficient to express many biologically relevant restrictions on the admissible update functions. To show a few examples, consider:

- *i*th input is essential in *f*:

$\mathrm{essentia}l_{i}(f):=\exists x\in B^{n}.f(x[i\;\mapsto \;0])⊕f(x[i\;\mapsto \;1])$ ;
- *f* is positively monotone in its *i*th input:

$\mathrm{positiv}e_{i}(f):=\forall x\in B^{n}.f(x[i\;\mapsto \;0])\Rightarrow f(x[i\;\mapsto \;1])$ ;
- *f* is negatively monotone in its *i*th input:

$\mathrm{negativ}e_{i}(f):=\forall x\in B^{n}.f(x[i\;\mapsto \;1])\Rightarrow f(x[i\;\mapsto \;0])$ .

We provide more examples of properties in the supplementary material. In addition to the positive and negative monotonicity of a specific input within a Boolean function *F_i_*, we describe the fact that *F_i_* is a ‘canalizing function’ (Harris et al. 2002) or a ‘veto function’ (Ebadi and Klemm 2014). Note that as the properties are defined in first-order logic, we can combine them with logical operators and customize them to only apply under some restrictions (e.g. *F_i_* is monotonous in input *j* only when input *j* is canalizing, etc.).

We can evaluate the properties on the update functions of a classical BN, and we get a yes/no answer, i.e. $\mathrm{essentia}l_{i}(F_{j})$ is true iff $v_{i}\in \mathrm{dep}(F_{j})$ etc. The evaluation can be extended to partially specified Boolean expressions, which we denote by prop[E] where prop is a property and *E* a partially specified Boolean expression. The outcome of such a property evaluation is the set of all interpretations for which the resulting Boolean function satisfies the property, e.g. $\mathrm{positiv}e_{i}(E_{j})$ is the set of all interpretations that ensure that (*v_i_*, *v_j_*) is an activation regulation. Formally, prop[E]={I|prop(E(I)) is true}.

The update function properties (UFP) of a PSBN are thus given as a set Π of Boolean function properties applied to partially specified Boolean expressions. The semantics of this is the set of all interpretations for which all the properties are satisfied, i.e. the intersection of the property evaluations. We say that an interpretation I is consistent with Π, if it belongs to this set. We further say that a BN $F^{\left(n\right)}$ is consistent with a PSBN $E^{\left(n\right)}$ and its update function properties Π if there exists an interpretation I consistent with Π such that F=E(I).

Note that the approach we take here, i.e. defining properties as intersections, is chosen for simplicity. A more general approach can be defined by using arbitrary (Boolean) combinations of property evaluations.

An example of update function properties is given in Fig. 2c. We require *v*_3_ to be essential and with negative monotone effect in *E*_1_, which effectively means that the only valid interpretation of ***f*** is the negation function. We furthermore require that both *v*_1_ and *v*_2_ be essential in *E*_3_ with *v*_1_ having a positive monotone effect, thus ensuring that the interpretations of ***h*** are limited to one of the four options: $v_{1}\vee v_{2},\;v_{1}\wedge v_{2},\;v_{1}\vee \neg v_{2},\;v_{1}\wedge \neg v_{2}$. The number of consistent BNs has now decreased to 16 (four options for ***h*** times four options for ***g***).

#### 3.1.4 Dynamic properties

In addition to properties of update functions, we typically consider other requirements and assumptions about the system behaviour represented as runs in the corresponding STG. Examples are properties of the attractor landscape (attractor multiplicity and type, phenotype expression, etc.) or other more general properties (basins of attraction, commitment sets, etc.).

To capture a wide variety of possible properties of runs, we rely on a hybrid extension of the branching-time temporal logic CTL (HCTL for short). Aside from standard Boolean connectives, HCTL consists of temporal operators that allow reasoning about the evolution of the system with respect to time and hybrid operators that quantify and reference individual model states. The most common such quantifier is the down-arrow operator, introduced in Goranko (1994, 2000) (denoted ↓), that binds a state variable to the current state.

Our presentation of HCTL follows Kernberger and Lange (2020). For additional details, such as the formal semantics of individual operators, see the supplementary material. Now, let AP be a non-empty finite set of atomic propositions and ${\text{Var}}_{s}$ be a countable set of state variables. The syntax of an HCTL formula is given by the following grammar, where *p* ranges over AP and *x* over ${\text{Var}}_{s}$:


$$\varphi ::=0|1|p|x|\neg \varphi |\varphi \wedge \varphi |{@}_{x}\;\;\varphi |↓x.\;\varphi |\exists x.\;\varphi |EX\varphi |E[\varphi U\varphi ]|A[\varphi U\varphi ].$$

The temporal operators EX, EU and AU have their usual intuitive meaning derived from CTL. We also employ the commonly used syntactic abbreviations for EF, AF, EG, AG, and AX (see supplementary material for details), as well as other first-order and propositional abbreviations (∀, ∨, ⇒, etc.). The intuition behind the hybrid operators is the following: The ‘at operator’ ${@}_{x}\;\;\varphi$ means “continue evaluating φ at the state *x*” while the ‘bind operator’ ↓x. φ stands for “name the current state of evaluation *x* and proceed to check φ under this assumption”. Overall, keep in mind that (as opposed to CTL), the validity of an HCTL formula generally also depends on some valuation of the state variables (${\text{Var}}_{s}\to B^{n}$).

The use of HCTL (as opposed to CTL) is motivated by the need to encode general properties of STG(F) without tying their validity to specific atomic propositions. For example, CTL alone cannot describe the general property of being a member of an attractor or having the ability to reach two distinct attractors (bi-stability). Meanwhile, the following hybrid formulae easily describe these two conditions in HCTL:


$$\begin{array}{c} \varphi_{1}=↓x.\;\mathrm{AGEF}x\\ \varphi_{2}=\exists a.\;\exists b.\;(EFa\wedge EFb\wedge ({@}_{a}\;\;\varphi_{1})\wedge ({@}_{b}\;\;(\varphi_{1}\wedge \neg EFa))). \end{array}$$

Guaranteeing such commonly expected biological properties in the candidate networks necessitates the use of hybrid operators. Furthermore, many of such temporal properties can be derived directly from observed data in real-world experiments (see the supplementary material). Finally, note that the CTL fragment of HCTL can be sufficient for many practical applications. Alternatively, a different temporal logic may be used altogether. Different variants of the BN sketch could be thus considered, relying on a modified notion of dynamic properties (DPs). A BN is consistent with a given DP if the property is satisfied by the respective STG.

An example of a DP is given in Fig. 2d. The formula, similar to $\varphi_{2}$ above, states that there exists a state (denoted by *c*) from which we can reach two different attractors. This represents a situation where two attractors share a (weak) basin. The number of BNs consistent with this property has now decreased to 4 (the remaining 12 BNs contain only a single attractor).

#### 3.1.5 Encoding of experimental data

So far we have considered those parts of the sketch that deal with prior knowledge (this is called an initial sketch). To incorporate reverse inference from experimental data, the binarized data are encoded using HCTL. In this way, we can regard the encoding of experimental data as a specific DP. This approach allows the combination of the forward and reverse inference in a single consistency checking procedure. A sketch extended with data encoded in this way is called a data-informed sketch.

We give the technical details of the procedure for building the HCTL formula for various kinds of data in the supplementary material. Here, we demonstrate the basic idea in a simple example. Suppose we are given the following binarized steady-state set:

**Table:** 

experiment	*v*_1_	*v*_2_	*v*_3_
1	0	0	1
2	1	1	0

The corresponding HCTL formula that expresses that the states (0, 0, 1) and (1, 1, 0) belong to some attractor is given in Fig. 2e. Notice that in this particular example, we finally obtain a single candidate BN—the interpretation of ***g*** a ***h*** is the following: $g=v_{1}$ and $h=v_{1}\wedge \neg v_{2}$. In general, however, the method may produce more candidate networks.

### 3.2 Inference problem

A BN sketch S is the tuple $S^{\left(n\right)}=(I^{\left(n\right)},E^{\left(n\right)},\Pi ,\Omega )$. Here, $I^{\left(n\right)}$ is an influence graph, $E^{\left(n\right)}$ is a PSBN, Π is the set of update functions properties (UFP), and Ω is a set of HCTL formulae, containing both the formulae describing the prior knowledge about the DPs, and the formulae encoding the experimental data. The formulae in Ω must not contain free variables. We say *F* is consistent with the sketch S if it is consistent with all its parts as defined in the previous.

**The BN inference problem:** Given a BN sketch S, our goal is to compute the set of all BNs consistent with S.

Note that if we solve the inference problem, we are also able to answer various queries. such as realizability “Is the set of consistent BNs non-empty?” or counting “How many consistent BNs exist?”

### 3.3 BN inference procedure

Our method utilizes symbolic representation by BDDs to concisely encode the set of candidate BNs that are potentially consistent with a particular sketch. Here, each candidate is encoded as a vector of Boolean values. A set of candidates can then be viewed as a Boolean formula (encoded via a BDD) satisfiable exactly by the members of the set. This set is gradually refined using individual constraints until only the consistent networks remain.

Algorithm 1: High-level BN inference procedure.

 **Input:** a BN sketch S=(I,E,Π,Ω)

 **Output:** a set *C* of candidate BNs

1 **if**E violates I**then reject**

2 E′← EliminateUninterpretedSymbols (E)

3 $\Pi \prime \leftarrow \;\mathrm{Substitute}(\Pi ,(E_{1},\ldots ,E_{n}),(E\prime_{1},\ldots ,E\prime_{n}))$

4 C← BddEncode(Π′)

5 **if**C is unsat**then reject**

6 G← ColouredSTG(E′)

7 $C\prime \leftarrow \;\mathrm{ColouredModelChecking}(\Omega ,G,B^{n}\times C)$

8 **if**C′ is unsat**then reject**

9 return C′

Overall, the inference procedure is summarized in Algorithm 1. Technical details regarding the implementation of procedures used within Algorithm 1 are then given in the supplementary material. Here, we merely provide a high-level overview of the whole method.

*Influence graph consistency checking (Line 1).* As the first step, we verify that the PSBN E is in agreement with the influence Graph I. This is performed on a syntactic level, checking that each expression *E_i_* only depends on the network variables *v_j_* for which $(v_{j},v_{i})\in I$.

*Uninterpreted function elimination (Lines 2, 3)*. As the second step, we eliminate the uninterpreted function symbols $f_{j}^{\left(a_{j}\right)}$ appearing in *E* for which $a_{j}>0$. Here, each $f_{j}^{\left(a_{j}\right)}$ is substituted with a logically equivalent expression using $2^{a_{j}}$ fresh, zero-arity symbols (constants) that together encode the truth table of the original $f_{j}$. This means that E′ only contains constant uninterpreted symbols. Let us refer to the set of the constant symbols as F′. On Line 3, we then substitute each *E_i_* within Π for the modified expression $E\prime_{i}$, i.e. each $\mathrm{prop}[E_{i}]$ is replaced by $\mathrm{prop}[E\prime_{i}]$.

*Properties encoding and validation (Lines 4, 5)*. Due to the previous step, the properties of Π′ now only contain constant uninterpreted symbols. As the properties are written in the first-order logic over Booleans, all the operations and quantifications in them can be implemented as BDD transformations. We can thus encode each $\mathrm{prop}[E\prime_{i}]$ as a BDD, whose variables correspond to the uninterpreted constants of $E\prime_{i}$. The whole Π′ is then constructed as an intersection of all these BDDs, which is again a BDD, denoted in the algorithm by *C*. If *C* represents an empty set, this means that the update function properties are contradictory, and the PSBN is thus not realizable.

*DPs validation (Lines 6, 7, 8)*. At this point, *C* encodes a set of candidate networks *F* that are consistent with the provided *I*, *E*, and Π. Based on this set, we can create a ‘coloured’ asynchronous state-transition graph *G* that collectively encodes every possible STG(F) corresponding to the networks within *C*. Using a bottom-up coloured model-checking procedure (see the supplementary material) operating on *G*, we can then further restrict *C* to C′, that guarantees the satisfaction of all the HCTL formulae in Ω encoding the DPs. During the intermediate steps of the procedure, the symbolic BDD encoding is extended to incorporate the state variables ${\text{Var}}_{s}$. However, since the formulae in Ω have no free variables, these do not appear in the resulting C′.

We should note that to actually obtain a candidate network from the set C′, a satisfying valuation of variables appearing within this BDD is mapped back to an interpretation I′ of the set F′ (appearing within E′), and subsequently to a network E′(I′). If an interpretation of the original F (appearing in *E*) is desired, this too can be constructed based on I′. By reversing the procedure, we can also check whether a given specific BN *F* is contained within the set C′.

Finally, let us make some remarks about the computational complexity of the algorithm. The dominating step of the algorithm is the coloured model checking (Line 7). As the algorithm is symbolic, its complexity has two components: first, the number of symbolic steps performed, and second, the complexity of the symbolic steps themselves.

The number of symbolic steps is bound by O(|S|×|φ|), although typically it is much smaller (see supplementary material for details). The performance of the individual symbolic steps then correlates with the size of the symbolic representation, which in our case depends on the number of interpretations. This number is in the worst case doubly exponential w.r.t. the arity of the function symbols. Such arity is then bounded by the in-degree (no. of incoming edges) within the influence graph. As argued in Kauffman (1969), the in-degree within BNs that exhibit complex behaviour is on average small (2–3 regulations), and this prediction appears to largely translate to real-world networks (Kadelka et al. 2020).

## 4 Evaluation and results

In this section, we demonstrate the applicability and scalability of our approach. For the applicability demonstration, we consider two case studies focussing on real biological models and data. In addition, we show the process of refinement of the sketch. The scalability of our method is then demonstrated on a set of large biological networks and synthetic steady-state data. Here, we present the main results of our experiments. Detailed information (including, e.g. particular HCTL formulae) is presented in the supplementary material. All experiments have been performed on a standard workstation with an 11th Gen Intel i5 CPU and 16 GB RAM. The prototype implementation and the results are available at https://github.com/sybila/boolean-network-sketches.

The first case study focuses on the T cell survival mechanism arising in the context of LGL leukaemia. The signalling network and a Boolean model characterizing this mechanism has been first designed by Zhang et al. (2008). Subsequently, in Saadatpour et al. (2011), the authors have developed a reduced version of the model. We consider the reduced variant. The reduced model contains 18 variables. One of them, called ‘Apoptosis’, is used to represent the programmed cell death.

The authors of the original Boolean model focussed on deducing the precise form of update functions from the literature. Such a task is often extremely difficult, since the existing data may be incomplete or imprecise, and may introduce certain inaccuracies or biases into the model. We show how these problems can be avoided by employing the inference approach based on network sketches. In particular, we consider two iterations of the inference procedure introduced in Section 3.3. In the first step, we address the question of whether there exists a consistent candidate. To that end, we incorporate the knowledge obtained from the existing signalling network and the binarized experimental data [taken from Saadatpour et al. (2011)]. Using the results of the first step, we then further refine the sketch and obtain the final results.

The existing signalling network includes two levels of prior knowledge—the influence graph *I* and the additional information about the influences (inhibition or activation). Using these characteristics, we generate the set Π of update function properties expressing the monotonicity of the respective influences. At this stage, we consider a BN *E* with completely unspecified update logic. To obtain a desired DP, we use the binarized experimental data addressing the state of several proteins observed under LGL leukaemia phenotype. Based on this data, we automatically generate a formula $\varphi_{1}$ encoding the existence of an attractor that contains a state corresponding with the data. The set of DPs Ω is then defined as $\Omega =\{\varphi_{1}\}$.

Next, we run the inference procedure on the complete sketch S=(I,E,Π,Ω). The whole computation takes <9 min. We find out that there exist consistent candidates. Individual rows of the second column of Table 1 show the number of candidates consistent with the particular components of the sketch *S*.

**Table 1.** Numbers of candidates consistent with the two sketches of the T cell survival model described in the first case study.

Sketch components	*S*	S′
IG	3.2e32	3.2e32
IG + PSBN	3.2e32	7.2e16
IG + PSBN + UFP	7.8e10	1296
IG + PSBN + UFP + DP	9.1e9	378

Each row shows the number of candidates consistent with the particular components of the sketch, starting with just the influence graph in the first row, and considering the whole sketch in the last row. Note that this corresponds to the Algorithm 1, where the sketch components are considered gradually.

In order to refine the sketch, we analyse the set of consistent networks. Our set representation allows us to symbolically compute attractors for all consistent candidates at once. By analysing this set of attractors, we observe two important things. First, there are candidates that do not exhibit any attractor corresponding directly to the programmed cell death phenotype. Second, some candidates do exhibit attractors that contain states where both the ‘Apoptosis’ variable and the variables for the various proteins are activated at the same time. However, once the programmed cell death process begins, the production of all proteins should cease.

We address the first issue by designing another DP, $\varphi_{2}$, that encodes the existence of a fixed-point attractor where the ‘Apoptosis’ variable is activated while all other variables (representing proteins) are deactivated. To address the second issue, we design an additional formula $\varphi_{3}$ encoding the DP expressing the observation there should not be any other attractor apart from those that correspond to the programmed cell death or the experimental data. We thus obtain a new set of DPs $\Omega \prime =\{\varphi_{1},\varphi_{2},\varphi_{3}\}$. Furthermore, we improve the specification of the update logic by substituting the component *E* of the sketch for a new (“more detailed”) PSBN E′. We require that when the ‘Apoptosis’ variable is activated, all other variables should be switched off. Therefore, the update function of each network variable *v_i_* (except ‘Apoptosis’) should be $\neg \mathrm{Apoptosis}\wedge f_{i}^{\left(a\right)}$, where *a* is a number of the variable’s regulators excluding ‘Apoptosis’, and $f_{i}^{\left(a\right)}$ represents an uninterpreted Boolean function with these *a* regulators as its arguments.

When we employ the new refined sketch S′=(I,E′,Π,Ω′) and run the inference algorithm, only 378 potential consistent networks remain. The whole computation for S′ only takes <1 s. The computation is an order of magnitude faster than the computation for *S* mainly because the searched space of candidates got notably smaller by substituting *E* for E′. Individual rows of the third column of Table 1 show the number of candidates consistent with the particular components of the sketch S′. By means of automatic analysis, we discover that all consistent candidates agree on the update functions for 13 variables (i.e. for each of these variables, only one consistent update function is possible). Modellers can use this information and only focus on the remaining five network components that vary among the candidates.

In our second case study, we show how network sketches can be useful for the development of the model of sepal primordium polarity for *Arabidopsis thaliana* (La Rota et al. 2011). We use the signalling network from the original article to obtain the influence graph and the update function properties. We again use a sketch with completely undefined update logic and determine DPs based on two expected attractor state (see the supplementary material for details). Since a case study regarding this model was also performed by the authors of the inference tool Griffin (Muñoz et al. 2018), we compare the performance of our method to theirs. We show that when we consider the exact same literature-based knowledge and data, both methods reach the same results. However, thanks to our symbolic representation, our computation is ∼50 000× faster, even though we consider more complex asynchronous semantics.

Finally, to further assess the scalability, we have tested our method on a set of complex sketches derived from large real-life models and synthetically generated steady-state data. We were able to successfully run the inference algorithm on models with up to 321 network variables. The employed sketches involve a significant amount of unknown information—their PSBN components admit up to 2^326^ candidate networks. Note that the computation times do not exceed 10 min even for the largest considered models. In the supplementary material, we present the detailed information regarding the methodology and the models used.

## 5 Discussion

In this article, we have introduced BN sketches as the original framework for fully automated inference of BN models from a combination of prior knowledge, experimental measurements, and additional biological hypotheses. The versatile formalism of partially specified update functions allows capturing partial knowledge of the update logic in a very general form, not restricted to a small set of patterns as used in other approaches.

The properties of the dynamic behaviour of the inferred model are described in a powerful formal language based on temporal logics allowing us to sufficiently express complex dynamic phenomena. The technical core of our approach is based on a very efficient formal method (coloured model checking) that guarantees to obtain the exact set of all candidate models that are in conformance with the knowledge specified in the sketch.

On the computational side, another advantage of our method is that we obtain a compact symbolic representation of the set of candidate models. This representation allows the user to easily obtain an arbitrary number of candidate BNs, which can be further analysed. The knowledge obtained during the analysis can be thus used to enrich the sketch for a subsequent iteration of the inference procedure.

In the evaluation, we have shown that our approach is practical and applicable to real-life cases. We have demonstrated that it scales even to large models. Experimentation with the method has revealed an important aspect of our workflow, namely the ability to gain insights from the iterative process of BN inference and use it to formulate additional hypotheses for the next iteration.

## Supplementary Material

btad158_Supplementary_Data
