# Supplementary material for: Boolean network sketches: a unifying framework for logical model inference
Source: Bioinformatics. 2023 Apr 2;39(4):btad158. doi: 10.1093/bioinformatics/btad158 (PMC10122605; doi:10.1093/bioinformatics/btad158)
Supplement: btad158_Supplementary_Data [file btad158_supplementary_data.pdf]

Boolean Network Sketches: A Unifying Framework for Logical Model Inference (Supplementary Material)

This document extends the main paper with the following information: Section 1 provides examples of update function properties together with intuitive explanation of their meaning. Section 2 formally describes the syntax and semantics of hybrid computation tree logic (HCTL) and shows how biological observations can be formulated in this logic. Section 3 gives a detailed description of the Boolean network inference algorithm that lies at the core of our method. Section 4 presents detailed information about the case studies used in the evaluation. Finally, Section 5 compares our approach to two related approaches; most notably, we show how these approaches are subsumed by our method.

1 Examples of Update Function Properties

Essentiality We want to specify that a specific variable (say, v_i) represents an *essential* input in the update function for v_j. Intuitively, this posits that there is at least one state in which v_i influences the outcome of the update function for v_j. We define:

$$essential_i(f) := \exists x \in \mathbb{B}^n . f(x[i \mapsto 0]) \oplus f(x[j \mapsto 1]).$$

Formally, the formula states that there are two states, sharing the values of all the variables except for v_i, for which the output of the update function is different. The desired property is then written as $essential_i[E_j]$.

Monotonicity Similarly, we can express the property of (positive or negative) input monotonicity. Intuitively, if v_i regulates v_j positively, an increase (from 0 to 1) in v_i can only result in an increase in v_j (however, v_j can also stay the same). For negative influence, the effect is reversed. We thus define:

$$positive_i(f) := \forall x \in \mathbb{B}^n.\ f(x[i \mapsto 0]) \Rightarrow f(x[i \mapsto 1])$$
$$negative_i(f) := \forall x \in \mathbb{B}^n.\ f(x[i \mapsto 1]) \Rightarrow f(x[i \mapsto 0]).$$

Formally, we require that for *every* pair of states that only differ in the value of v_i, our desired implication is satisfied.

Canalising functions Other potentially useful update function properties include *canalising* functions [6]. Intuitively, a function is canalising if it is biased with respect to a specific input variable. A specific value of a particular input guarantees a specific output value. We define:

$$\mathit{sufficient}_{i,b,o}(f) := \forall x \in \mathbb{B}^n.\ f(x[i \mapsto b]) \Leftrightarrow o.$$

to mean "$v_i = b$ is sufficient for output o". We may then specify properties such as $\mathit{sufficient}_{i,1,0}[E_j]$ meaning "$v_i = 1$ is sufficient for the update function represented by E_j to be false".

Veto functions A veto function [5] is *true* only when all its negative inputs are *false* and at least one of its positive inputs is *true* (the definition assumes all inputs are monotonic). When all inputs are also essential, such condition corresponds to a single update function that can be written out explicitly. However, if some of the inputs remain non-essential, we have to formulate the condition as a combination of update function properties.

To enforce a veto update function of variable v_i with possibly non-essential inputs, we assume two uninterpreted function symbols $\boldsymbol{f_P}^{(q)}$ and $\boldsymbol{f_N}^{(r)}$, such that $E_i = \boldsymbol{f_P}(v_{p_1}, \ldots, v_{p_q}) \wedge \boldsymbol{f_N}(v_{n_1}, \ldots, v_{n_r})$ where v_{p_j} are the positive and v_{n_k} the negative inputs of the update function for v_i. We define:

$$\begin{aligned} \mathit{veto}_{p_1,\ldots,p_q;n_1,\ldots,n_r} := & \bigwedge_{j=1}^{q} \Big(\mathit{essential}_{p_j}(f) \Rightarrow \mathit{sufficient}_{p_j,1,0}(f)\Big) \\ & \wedge \bigwedge_{k=1}^{r} \big(\mathit{essential}_{n_k}(f) \Rightarrow \mathit{sufficient}_{n_k,1,1}(f)\big)\,. \end{aligned}$$

The desired property is then written as $\mathit{veto}_{p_1,\ldots,p_q;n_1,\ldots,n_r}[E_i]$.

Intuitively, the property states that if v_{n_k} is an essential input of the update function, then the value of the function has to be *false* whenever v_{n_k} is *true* (no essential negative regulator can be *true* in order for the update function to be *true*). Furthermoe, whenever a value of some essential input v_{p_j} is *true* (and the condition for negative regulators is satisfied), the output of the update function has to be *true* as well.

Other properties The case of veto functions nicely illustrates how multiple properties can be combined to enforce the input-output behaviour of more complex functions. There are numerous other combinations of properties that can be tailored to fulfil the specific needs of a particular biological process. One example could be conditional essentiality, where we assume an input is essential only when additional properties hold. Say that v_i activates v_j, but only when a specific catalyst v_k is present. We can provide a property that states that v_i is essential in v_j, but *only* for the cases where v_k is 1. In other words, v_i and v_k work in *cooperation*. Similarly, a notion of *antagonism* between function inputs could be formalised.

2 HCTL Syntax, Semantics, Examples

The presentation of the HCTL follows [7].

Definition 1 *Let AP be a finite set of* atomic propositions *and Var_{s} be a countable set of* state variables. *A* dynamic property *φ is a HCTL formula defined as follows:*

$$\varphi ::= p \mid x \mid \neg\varphi \mid \varphi \wedge \varphi \mid @_x\ \varphi \mid {\downarrow}x.\ \varphi \mid \exists x.\ \varphi \mid \mathbf{EX}\,\varphi \mid \mathbf{E}[\varphi\,\mathbf{U}\,\varphi] \mid \mathbf{A}[\varphi\,\mathbf{U}\,\varphi]$$

Here, p ranges over AP and x over Var_{s}.

Before we define the formal meaning of each operator, let us also observe that we use the standard abbreviations that define additional commonly used operators:

$$\begin{aligned}
\mathbf{AX}\,\varphi &\equiv \neg\,\mathbf{EX}\,\neg\varphi\\
\mathbf{EF}\,\varphi &\equiv \mathbf{E}[\mathit{true}\,\mathbf{U}\,\varphi]\\
\mathbf{AF}\,\varphi &\equiv \mathbf{A}[\mathit{true}\,\mathbf{U}\,\varphi]\\
\mathbf{EG}\,\varphi &\equiv \neg\,\mathbf{AF}\,\neg\varphi\\
\mathbf{AG}\,\varphi &\equiv \neg\,\mathbf{EF}\,\neg\varphi\\
\forall x.\ \varphi &\equiv \neg\exists x.\ \neg\varphi
\end{aligned}$$

We also use the usual propositional operators, such as $\vee$ (disjunction), $\Rightarrow$ (implication), $\Leftrightarrow$ (equivalence), $\oplus$ (non-equivalence, exclusive disjunction).

Semantics The validity of a dynamic property relates to the state-transition graph STG(F), not the BN F directly. Consequently, we define a *run* π to be a maximal sequence of states $\pi = \pi_1, \pi_2, \ldots$ satisfying $\pi_i \to \pi_{i+1}$ for every i. Note that every maximal run is necessarily infinite, as every state in STG(F) has at least one outgoing transition.

Additionally, we extend the state-transition graph STG(F) with a labelling function $L : \mathbb{B}^n \to 2^{AP}$ which assigns to each state a subset of atomic propositions that hold in said state. For the purposes of this paper, we consider that $AP = \{p_1, \ldots, p_n\}$ contains propositions that identify states where individual variables hold. That is, $p_i \in L(s)$ if and only if v_i is true in state s. However, additional biologically motivated propositions can be also included, for example identifying states corresponding to specific phenotypes.

We write $\mathcal{R}_{F,s}$ to denote the set of all runs of STG(F) starting in s (i.e. $\pi_1 = s$), $\nu : \mathrm{Var}_{\mathsf{s}} \to \mathbb{B}^n$ to denote a valuation of the state variables, and define the

satisfaction relation of a formula φ in a given state $s \in \mathbb{B}^n$ as follows:

$$
\begin{aligned}
(F,s,\nu) &\models p && \iff p \in L(s) \\
(F,s,\nu) &\models x && \iff \nu(x) = s \\
(F,s,\nu) &\models \neg\varphi && \iff (F,s,\nu) \not\models \varphi \\
(F,s,\nu) &\models \varphi_1 \wedge \varphi_2 && \iff (F,s,\nu) \models \varphi_1 \text{ and } (F,s,\nu) \models \varphi_2 \\
(F,s,\nu) &\models @_x\ \varphi && \iff (F,\nu(x),\nu) \models \varphi \\
(F,s,\nu) &\models \downarrow x.\ \varphi && \iff (F,s,\nu[x \mapsto s]) \models \varphi \\
(F,s,\nu) &\models \exists x.\ \varphi && \iff (F,s,\nu[x \mapsto s']) \models \varphi \text{ for some } s' \in \mathbb{B}^n \\
(F,s,\nu) &\models \mathbf{EX}\,\varphi && \iff (F,\pi_1,\nu) \models \varphi \text{ for some } \pi \in \Pi_{F,s} \\
(F,s,\nu) &\models \mathbf{E}[\varphi_1\,\mathbf{U}\,\varphi_2] && \iff \text{there exists } \pi \in \Pi_{F,s} \text{ and } i \in \mathbb{N} \text{ such that} \\
& && \quad\ (F,\pi_i,\nu) \models \varphi_2 \text{ and } \forall j < i.\ (F,\pi_j,\nu) \models \varphi_1 \\
(F,s,\nu) &\models \mathbf{A}[\varphi_1\,\mathbf{U}\,\varphi_2] && \iff \text{for all } \pi \in \Pi_{F,s} \text{ there exists } i \in \mathbb{N} \text{ such that} \\
& && \quad\ (F,\pi_i,\nu) \models \varphi_2 \text{ and } \forall j < i.\ (F,\pi_j,\nu) \models \varphi_1
\end{aligned}
$$

As before, $\nu[x \mapsto s]$ denotes a copy of the valuation ν where the variable x is mapped to the state s. When φ is closed (i.e. without free variables), the choice of ν is irrelevant and we may simply write $(F, s) \models \varphi$.

Consistency Given a *closed* HCTL formula φ describing a dynamic property as outlined above, we say that a Boolean network F is *consistent* with φ if we have $(F, s) \models \varphi$ for every $s \in \mathbb{B}^n$, i.e. all the states of STG(F) have to satisfy φ. Note that this comes without loss of generality—if we instead wanted to specify that there exists at least one state satisfying a given formula φ, we can rewrite the formula to $\exists x.\ @_x\ \varphi$. This new formula holds in all states if and only if the original formula φ holds in at least one state.

2.1 Biological Observations as Dynamic Properties

We now discuss how various types of real-world observations can be translated into dynamic properties that are incorporated into a Boolean network sketch. In the following, we consider various types of data and associated assumptions (partial observability, time series, attractors, knockouts, etc.). However, in all cases, we assume that the data has been already cleaned up and binarised using some standard tool. We do not address this aspect of the workflow explicitly, as it is largely orthogonal to our method.

Partial observations Dynamic properties allow us to reason about the evolution of the system's state with respect to discrete time steps. To this end, we need to formalise how the system's state is translated into a logical formula. In general, the system's state $s \in \mathbb{B}^n$ corresponds to the values of its n Boolean variables. In HCTL, each state can be thus described using a conjunction of n literals (atomic propositions or negated atomic propositions) corresponding to the values of variables within s.

However, in practice, even the most precise measurements may not include all n network variables. We thus generally assume that every measurement is potentially only an *incomplete* collection of observations. Formally, a partial observation o is a *partial* function $o : \{1, \ldots, n\} \nrightarrow \mathbb{B}$ which assigns Boolean values to some network variables. To capture such observation in a temporal logic, we again use a conjunction of literals. We write φ_o to denote the logical formula expressing the partial observation o:

$$\varphi_o \equiv \bigwedge_{o(i)=1} p_i \wedge \bigwedge_{o(i)=0} \neg p_i$$

The formula φ_o is then satisfied by all states that result in the corresponding observation o. So for example, if a partial observation fixes 6 out of 10 network variables, then there are 2^4 states that could result in this partial observation, and these satisfy the formula φ_o.

Time series A common representation of measurement data is a time series, represented by a sequence of partial observations, $o_1, \ldots, o_k$. Here, each o can potentially fix different network variables; for example, a measurement of a variable in some time step may have been discarded due to errors, while other time steps and variables are measured correctly.

The general assumption is that the partial observations are of the *same system* and were measured in a sequence. From the perspective of the system's discrete dynamics, this means that there should be a path in the system's state space that replicates the partial observations in the right order.

However, note that the states replicating each observation do not have to be direct successors on this path (as is often incorrectly assumed in literature, see [9, 12] for details). In fact, not all states on such a path must necessarily match some observation o_i either. In general, the system's behaviour is unknown while the system is not observed, and we thus avoid any assumptions in this regard. Due to the loss of time information by the discrete abstraction, we also cannot impose any specific timing properties (e.g. states being reachable within k steps).

To encode the presence of a time series in a system, we use the following formula:

$$\varphi_{o_1,\ldots,o_k} \equiv (\varphi_{o_1} \wedge \mathbf{EF}(\ldots(\varphi_{o_{k-1}} \wedge \mathbf{EF}\,\varphi_{o_k})))$$

This formula holds in every state from which we can replicate the observed time series. We can thus write $\exists x.\ @_x\ \varphi_{o_1,\ldots,o_k}$ to obtain a formula that holds if there is *some* way to replicate the given time series, starting in *some* model state.

Note that here, we allow the system to non-deterministically evolve into completely different outcomes (in addition to the specified time series). If there is sufficient evidence to support the claim that this particular time series is guaranteed to occur for some initial conditions (i.e. the system cannot diverge), we can replace **EF** with **AF** to further restrict the space of satisfying states to those that replicate the time series on *all* runs.

Time series with path properties We can also use the until operators (**EU** and **AU**) to incorporate properties that should be universally true along the duration of the time series. For example, say that φ_{alive} describes the states of the system which we assume are in some sense admissible for the observed experiment (e.g. they describe states in which the cell is still alive).

Now, in the context of $\varphi_{o_1,\dots,o_k}$, instead of $\mathbf{EF}\, o_i$, we can write $\mathbf{E}\, \varphi_{alive}\, \mathbf{U}\, \varphi_{o_i}$, which states that the observation o_i is not only reachable, but reachable by a *living* cell. Furthermore, each o_i can be possibly associated with a different path property. For example, if certain observations were taken in different phases of the cell cycle, we can express this using corresponding path properties. Using this mechanism, we can further restrict the satisfying networks only to those which are biologically admissible.

Periodic time series In some instances, the observed time series may represent a single period of an oscillation, with the assumption that once o_k is reached, o_1 should be eventually observed again and the whole time-series should be repeatable. This type of data may come for example from observing a cell cycle or a circadian rhythm. To describe time series that is periodically repeatable, we can then use the following formula:

$$\varphi_{(o_1,\dots,o_k)^+} \equiv \downarrow x.\ (\varphi_{o_1} \wedge \mathbf{EF}(\dots(\varphi_{o_{k-1}} \wedge \mathbf{EF}(\varphi_{o_k} \wedge \mathbf{EF}\, x))))$$

Note that this is the first time we use a hybrid operator ($\downarrow$) in this section. In this situation, the presence of the hybrid operator greatly simplifies the specification. If we only considered CTL, we could still describe a situation where the time series is repeatable finitely many times (by nesting the $\varphi_{o_1,\dots,o_k}$ formula), but there is no direct way to guarantee that it can be repeated indefinitely. However, using $\downarrow$, we clearly specify that the initial state of the time series has to be reachable from the final state.

Finally, we may again consider **AF** instead of **EF** if the time series must be reproducible repeatedly on *every* path starting in the satisfying state, and we can use **EU** or **AU** to implement path properties.

Attractor data In many practical instances, the systems under observation are assumed to be in the so called *steady state*. Here, it does not necessarily mean that every variable of the system has a single fixed value, but rather that the behaviour of the system is confined to a subset of states that it cannot leave without external intervention. Such situation corresponds to the notion of attractors in Boolean networks.

To specify that a particular observation o must be present in a system attractor, we can write the following:

$$\varphi_{A(o)} \equiv \downarrow x.\ \varphi_o \wedge \mathbf{AG}\, \mathbf{EF}\, x$$

Informally, this formula is satisfied in a state where φ_o holds, and on all paths leaving this state, we always have the possibility of returning. Such formula is

satisfied in any state that is a member of an attractor and reproduces the given observation o. We can use $\exists x.\, @_x.\, \varphi_o \wedge \mathbf{AG}\,\mathbf{EF}\, x$ to describe that such a state exists *somewhere* in the state space.

In general, we may also write $\varphi_A \equiv \downarrow x.\; \mathbf{AG}\,\mathbf{EF}\, x$ to denote a HCTL formula that is true in exactly all the attractor states of the system (in other words, every state that is reachable from x can also reach x back). We can then simplify the condition above to $\varphi_A \wedge \varphi_o$. Using this composition, we can express different properties that have to hold within the system's attractors. For example, we use $\varphi_A \wedge \varphi_{o_1,\ldots,o_k}$ to write that a particular time series must be reproducible within a system attractor (due to the nature of attractors, this also means that the time series can be repeated indefinitely).

3 Symbolic BN Inference Algorithm

The inference algorithm consists of the following steps:

1. influence graph consistency checking (line 1 in Algorithm 1),
2. building a coloured state-transition graph:
   (a) uninterpreted function elimination (lines 2, 3 in Algorithm 1),
   (b) update function properties validation (lines 4, 5 in Algorithm 1),
3. coloured HCTL model checking (lines 6, 7, 8 in Algorithm 1).

In the first step, we simply verify that the PSBN $E^{(n)}$ is in agreement with the influence graph I by checking that each partially specified Boolean expression E_i only depends on the network variables v_j for which $(v_j, v_i) \in I$. If this check fail, the input is invalid and the offending variables are reported to the user for consideration whether to add new edges to the influence graph or to eliminate the variables from the partially specified Boolean expressions.

3.1 Coloured state-transition graphs

To capture the semantics of a PSBN, which represents a collection of standard BNs, we are going to need the notion of a *coloured state-transition graph* (CSTG). This graph will then be used as the input to the model-checking core of our algorithm.

Definition 2 *A* coloured state-transition graph *is a triple* $(S, \mathcal{C}, T)$ *where* S *is a set of* states, $\mathcal{C}$ *is a set of colours, and* $T \subseteq S \times \mathcal{C} \times S$ *is a set of transition relations* $T = \{T_c \mid c \in \mathcal{C}\}$ *such that each* $T_c \subseteq S \times S$.

A coloured state-transition graph is thus a representation of a family of state-transition graphs whose set of states is shared, but which can have distinct transition relations. This notion maps well to our need of capturing the semantics

of PSBN, which can be seen as a family of Boolean networks with the same set of variables but possibly differing update functions.

Technically, in order to build the CSTG, we first need to convert the partially specified Boolean expressions of the given PSBN into an equivalent form that only uses 0-arity uninterpreted function symbols (i.e. constants). For each $\boldsymbol{f}^{(a)}$, we create 2^a fresh uninterpreted constant symbols and substitute all occurrences of $\boldsymbol{f}$ with a logically equivalent expression using these fresh symbols.

For a function symbol $\boldsymbol{f}$ of arity one, the expression $\boldsymbol{f}(e)$ (where e is an arbitrary Boolean term) is replaced by $(e \Rightarrow \boldsymbol{c_1}) \wedge (\neg e \Rightarrow \boldsymbol{c_2})$, where $\boldsymbol{c_1}$ and $\boldsymbol{c_2}$ are fresh constant symbols. For a function symbol $\boldsymbol{f}$ of arity two, the expression $\boldsymbol{f}(e_1, e_2)$ (where e_1, e_2 are arbitrary Boolean terms) is replaced by

$$((e_1 \wedge e_2) \Rightarrow \boldsymbol{c_{1,1}}) \wedge ((e_1 \wedge \neg e_2) \Rightarrow \boldsymbol{c_{1,2}}) \wedge ((\neg e_1 \wedge e_2) \Rightarrow \boldsymbol{c_{2,1}}) \wedge ((\neg e_1 \wedge \neg e_2) \Rightarrow \boldsymbol{c_{2,2}})$$

where $\boldsymbol{c_{1,1}}$, $\boldsymbol{c_{1,2}}$, $\boldsymbol{c_{2,1}}$, $\boldsymbol{c_{2,2}}$ are fresh constant symbols. Similar constructions are done for higher arities.

We denote the set of all the new constant uninterpreted symbols by $\mathcal{F}'$ and the expressions created from E_i by the above substitution by E'_i for all i. We also modify the set of update function properties Π by replacing all $prop[E_i]$ with $prop[E'_i]$ and call this new set of update function properties Π'.

The CSTG is then build as follows: The set of states $S = \mathbb{B}^n$ as in the (standard) BN case. The set of colours $\mathcal{C}$ is the set of all interpretations consistent with the update function properties Π'. In the following, we speak about colours instead of interpretations and use lowercase letters such as c to refer to specific colours. The set of transition relations T is defined to be the set of all transition relations T_c such that T_c is the transition relation of the Boolean network created from the PSBN by fixing the interpretation c.

Note that we assume that both the set of states and the set of transitions can be represented symbolically. In our implementation, we use the formalism of BDDs, but the algorithm can work with other symbolic representations.

3.2 Coloured HCTL model checking

The core of our algorithm is a model-checking procedure that takes as an input: a coloured state-transition graph, a state labelling function, and a HCTL formula. The CSTG has been build in the previous part. The labelling function we use is defined in Section 2. Finally, the HCTL formula is obtained by taking a logical conjunction of all the formulae in Ω. We call the resulting formula φ.

In the following, we use Var_φ to denote the (finite) set of state variables that appear in the formula φ. We use the notation S^{Var_φ} for the set of all functions $Var_\varphi \rightarrow S$, i.e. the set of all assignments of states to the variables of Var_φ. Before we introduce the algorithm itself, we briefly discuss the assumptions about the symbolic description of the inputs, including the symbolic operations used in the algorithm.

3.2.1 Symbolic Computation Model

The basic building blocks of our algorithm are certain symbolic steps, set and relational operations that can be performed over a given symbolic representation of the state space. Because we need to reason about both the colours and the valuation of the state variables, we work with the set $S \times \mathcal{C} \times S^{Var_\varphi}$, which can also be seen as a relation of arity $(|Var_\varphi| + 2)$. We sometimes call this set the *extended state space* and its elements *extended states*. We assume that this set and its arbitrary subsets can be represented symbolically. The symbolic operations we use in our algorithm are described in Table 1. Note that the extended state space manipulation operators from Table 1 can usually be implemented in terms of basic set and relation algebra.

Intuitively, the role of the *existential quantification* operations is to "forget" certain information about the current subset of the extended state space: $\exists_{st}$ forgets the current state while $\exists_x$ forgets the valuation of the state variable x. The *equaliser* of x, $\textsc{Eq}(x)$ is then a subset that holds a single piece of information, namely that the valuation of the state variable x should be equal to the current state. Finally, the most important operation is the *coloured pre-image*, $\textsc{Pre}(X)$. Given a subset of the extended state space, this operation computes the set of all predecessors respecting the colours of the transitions while keeping the valuation of the state variables intact.

We assume that every operation in Table 1 has a constant symbolic complexity. This is a fairly standard assumption for the analysis of symbolic algorithms like ours. However, in practical cases, it may actually be preferred to implement some of these operations as multiple, less resource-intensive symbolic steps. The *saturation* method [4] is a typical example of this phenomenon.

3.2.2 Coloured HCTL model-checking algorithm

The pseudocode of the algorithm is given as Algorithm 1. As in standard symbolic CTL model checking, we assume that the formula is given in a normal form that only uses the temporal operators **EX**, **EG**, and **EU**. This assumption comes without any loss of generality due to the following equivalence of formulae:

$$\mathbf{A}[\varphi_1 \, \mathbf{U} \, \varphi_2] \equiv \neg \, \mathbf{EG} \, \neg\varphi_2 \wedge \neg \, \mathbf{E}[\neg\varphi_2 \, \mathbf{U}(\neg\varphi_2 \wedge \neg\varphi_1)]$$

This equivalence holds in standard CTL as well as in its hybrid extension. Note that although the right-hand-side formula is larger, parts of it representing the sub-formula φ_2 are shared. Due to the dynamic programming (memoization) nature of the algorithm, as explained below, this does not lead to any blowup in the actual computation.

The algorithm begins by computing the set Var_φ of variables that appear in the given formula. Once the set Var_φ is computed, it is considered a global constant during the rest of the computation, together with the input CKS. The rest of the algorithm proceeds by recursively calling the $\textsc{Check}$ function. This function accepts a sub-formula of the main formula as its input and produces a set of extended states (s, c, ρ) such that the state s satisfies the given sub-formula

Table 1: Summary of symbolic operations appearing in the presented algorithm. The description assumes a CKS $\mathcal{K} = (S, \mathcal{C}, T, L)$ and a finite set Var_φ containing exactly all the variables appearing in the HCTL formula φ to be checked. The extended state space manipulation operations can be implemented using the standard and relational operations.

Standard set operations		
union	$A \cup B$	$\{x \mid x \in A \vee x \in B\}$
intersection	$A \cap B$	$\{x \mid x \in A \wedge x \in B\}$
difference	$A \setminus B$	$\{x \mid x \in A \wedge x \notin B\}$
product	$A \times B$	$\{(x, y) \mid x \in A \wedge y \in B\}$
Extended state space manipulation ($X \subseteq S \times \mathcal{C} \times S^{Var_\varphi}$)		
existential quantification of the state	$\exists_{st}(X)$	$\{(s', c, \rho) \mid s' \in S \wedge \exists s : (s, c, \rho) \in X\}$
existential quantification of a state variable	$\exists_x(X)$	$\{(s, c, \rho') \mid \rho' \in S^{Var_\varphi} \wedge \exists \rho : (s, c, \rho) \in X \wedge \forall y \in Var_\varphi \setminus \{x\} : \rho'(y) = \rho(y)\}$
equaliser of the state and a state variable	$\textsc{Eq}(x)$	$\{(\rho(x), c, \rho) \mid c \in C \wedge \rho \in S^{Var_\varphi}\}$
coloured pre-image	$\textsc{Pre}(X)$	$\{(s, c, \rho) \mid \exists t : (t, c, \rho) \in X \wedge (s, t) \in T_c\}$

in the Kripke structure $\mathcal{K}_c$ with ρ as the state variable valuation. For efficient computation, the function is supposed to be memoized, i.e. its results should be cached and used whenever the same sub-formula is to be processed.

The computation of the Check function is mostly straightforward. The algorithm for **EU** comes from the fact that $\mathbf{E}[\psi_1 \, \mathbf{U} \, \psi_2] \equiv \psi_2 \vee (\psi_1 \wedge \mathbf{EX} \, \mathbf{E}[\psi_1 \, \mathbf{U} \, \psi_2])$ and that the **EU** operator is the least fixed point of this equation. Similarly, the algorithm for **EG** comes from the fact that $\mathbf{EG} \, \psi \equiv \psi \wedge \mathbf{EX} \, \mathbf{EG} \, \psi$ and that the **EG** operator is the greatest fixed point of this equation. The proof of these claims in HCTL is the same as for standard CTL.

As for the hybrid extension, to evaluate the sub-formula x where x is a state variable, we simply take the equaliser of x, as this sub-formula is true in exactly all the extended states where the valuation of x is the current state. To evaluate the *jump* operator $@_x.\psi$, we first compute all the extended states where ψ holds. Out of them, we filter only those extended states where the valuation of x is the current state. And finally, we "forget" the current state as the satisfaction of $@_x.\psi$ is independent of it. To evaluate the existential quantification $\exists x.\psi$, we again first compute $\textsc{Check}(\psi)$. Then, we simply "forget" the valuation of x as x is no longer a free variable in $\exists x.\psi$. Finally, to evaluate the *bind* operation $\downarrow x.\psi$, we do a computation similar to the previous case, but we filter only those states where the valuation of x is the current state.

Once the Check function computes the set of extended states for the whole

Algorithm 1: Coloured Symbolic model checking for HCTL

Input: $\mathcal{K} = (\mathcal{C}, S, T, L)$ is a coloured Kripke structure; φ is a HCTL formula

Output: the set $\mathcal{R}^{\mathcal{K}}_{\varphi} = \{(s,c) \in S \times \mathcal{C} \mid (\mathcal{K}_c, s) \models \varphi\}$

$Var_\varphi \leftarrow \{x \in \mathrm{Var} \mid x$ appears in $\varphi\}$
$V \leftarrow \textsc{Check}(\varphi)$
return $\{(s,c) \mid \exists \rho : (s,c,\rho) \in V\}$

Function $\textsc{Check}(\psi)$ *(memoized)*
  switch ψ **do**
    case $p \in AP$ **do return** $\{(s,c,\rho) \in S \times \mathcal{C} \times S^{Var_\varphi} \mid s \in L(p)\}$
    case $\neg\psi'$ **do return** $S \times \mathcal{C} \times S^{Var_\varphi} \setminus \textsc{Check}(\psi')$
    case $\psi'_1 \wedge \psi'_2$ **do return** $\textsc{Check}(\psi'_1) \cap \textsc{Check}(\psi'_2)$
    case $\mathbf{EX}\,\psi'$ **do return** $\textsc{Pre}(\textsc{Check}(\psi'))$
    case $\mathbf{E}[\psi'_1\,\mathbf{U}\,\psi'_2]$ **do**
      $B \leftarrow \textsc{Check}(\psi'_2)$
      $Set_{\psi'_1} \leftarrow \textsc{Check}(\psi'_1)$
      repeat $B \leftarrow B \cup (Set_{\psi'_1} \cap \textsc{Pre}(B))$ **until fixpoint**
      return B
    case $\mathbf{EG}\,\psi'$ **do**
      $B \leftarrow \textsc{Check}(\psi')$
      repeat $B \leftarrow B \cap \textsc{Pre}(B)$ **until fixpoint**
      return B
    case $x \in Var_\varphi$ **do return** $\textsc{Eq}(x)$
    case $\downarrow x.\psi'$ **do return** $\exists_x(\textsc{Eq}(x) \cap \textsc{Check}(\psi'))$
    case $@x.\psi'$ **do return** $\exists_{\mathrm{st}}(\textsc{Eq}(x) \cap \textsc{Check}(\psi'))$
    case $\exists x.\psi'$ **do return** $\exists_x(\textsc{Check}(\psi'))$

original formula φ, the algorithm discards the state variable valuation part of the set and returns the result. Note that as the whole formula is closed, the valuation plays no role in the final result.

The correctness of the algorithm follows from the discussion above, the observations about the equivalences of formulae, the fixed point properties, and the semantics of HCTL operators. The worst-case complexity of the algorithm in terms of the symbolic steps as described above is $\mathcal{O}(|S| \times |\varphi|)$. The only interesting cases complexity-wise are the computations of the fixed points. Note that as each invocation of Pre creates a set of predecessors, there cannot be more such invocations than the length of the longest simple path in the CKS. Clearly, such a length is less than the number of states. Note that the complexity is independent of $\mathcal{C}$ as well as of $|Var_\varphi|$. Although this might seem surprising, it is due to the fact that the Pre operation computes the predecessors in all colours and state variable valuations at once.

It is true, however, that the complexity of the symbolic steps themselves may vary depending on the size of the extended state space representation, which might even change during computation. The representation may depend on both the number of colours and the number of state variables in the formula. As explained in Section 3.1, the number of colours coresponds to the number of uninterpreted symbols and their arity. This correspondence is doubly exponential: a function symbol $\boldsymbol{f}$ of arity a is encoded with 2^a constant symbols. Such a symbol thus has 2^{2^a} interpretations in the worst case. Note that this number may, however, be reduced by the restrictions given by the update function properties.

On the other hand, the worst case (in the number of symbolic steps) only happens when all of the fixed-point computations in the algorithm explore the whole state space one state at a time. This is far from the expected case, as the Pre operation has the potential to discover larger chunks of the state space at once.

4 Evaluation

In this section, we present detailed information about our experiments.

4.1 Analysis of the T Cell Survival Network

In our first case study, we focus on the T cell survival mechanism arising in the context of T-LGL leukaemia. In [14], the authors have designed the signalling network and Boolean model characterising this mechanism. They have created the model based on an extensive literature search. Subsequently, in [11], the authors have developed a reduced version of that model, which we focus on in our evaluation. This model contains 18 variables. We call the set of these variables Var. Their names are shown in the first column of Table 2. One of them, called *Apoptosis*, is used to model programmed cell death.

When creating the original Boolean model, the authors had to deduce the precise form of update logic from the literature. Such a task is often extremely challenging, since the existing data may be incomplete or imprecise, and may result in introducing certain inaccuracies or biases into the model. We show how these problems can be avoided by employing the inference approach based on network sketches, which does not require a complete specification of the update logic. We also show the process of refinement of the sketch. Particularly, we consider two iterations of the inference procedure. In the first step, we address the question of whether there exists a consistent candidate. To that end, we incorporate the knowledge obtained from the existing signalling network and the binarised experimental data (both taken from [11]). Using the results of the first step, we then further refine the sketch and obtain the final results.

The existing signalling network [11] includes two levels of prior knowledge – the influence graph I and the additional information about the influences (inhibition or activation). Using these characteristics, we generate a set of update function properties expressing the monotonicity of the respective influences.

Variable	TLGL state
Apoptosis	OFF
BID	OFF
Caspase	OFF
Ceramide	OFF
CREB	?
CTLA4	?
DISC	OFF
Fas	OFF
FLIP	ON
GPCR	OFF
IAP	?
IFNG	OFF
MCL1	ON
P2	?
sFas	ON
SMAD	?
S1P	ON
TCR	?

Table 2: List of variables used in the model of T cell survival network [11]. The second column shows the state of several nodes observed under the LGL leukaemia phenotype, as defined in [11].

Moreover, we require all described regulations to be essential since the knowledge is based on literature (and not, e.g., some hypotheses). Combining the properties expressing the monotonicity and essentiality, we get the set Π. At this stage, we consider a BN E with completely unspecified update logic.

To obtain a desired dynamic property, we use the binarised experimental data [11] addressing the state of several proteins observed under the LGL leukaemia phenotype. The data concerns network components that were experimentally found to be overexpressed (resp. underexpressed). Moreover, variable *Apoptosis* should be inactive. We present the binarised values of measured variables in the second column of Table 2. Based on this data, we automatically generate a formula φ_1 encoding the existence of an attractor that contains a state corresponding with the data. The set of dynamic properties Ω is then defined as $\Omega = \{\varphi_1\}$.

$$\psi_{data} \equiv (\text{S1P} \wedge \text{sFas} \wedge \neg\text{Fas} \wedge \neg\text{Ceramide} \wedge \neg\text{Caspase} \wedge \text{MCL1} \wedge \neg\text{BID} \wedge \neg\text{DISC} \wedge \text{FLIP} \wedge \neg\text{IFNG} \wedge \neg\text{GPCR} \wedge \neg\text{Apoptosis})$$

$$\varphi_1 \equiv \exists x.\, @_x.\ (\psi_{data} \wedge \mathbf{AG}\,\mathbf{EF}(\psi_{data} \wedge x))$$

Combining the four components, we obtain the complete sketch $S = (I, E, \Pi, \Omega)$. We run the inference procedure on the sketch S, which takes less than 9 minutes. Individual rows of the second column of Table 3 show the number of candidates consistent with the particular components of the sketch S.

In order to refine the sketch, we analyse the set of consistent networks. Our set representation allows us to symbolically compute attractors for all consistent candidates at once. For this task, we use the method introduced in [1]. By analysing the computed attractors, we observe two important things. First, there are candidates that do not exhibit any attractor corresponding directly to the programmed cell death phenotype. Second, some candidates do exhibit attractors that contain states where both the *Apoptosis* variable and variables for various proteins are activated at the same time. However, once the programmed cell death process begins, the production of all proteins should cease.

We address the first issue by designing another dynamic property, φ_2, that encodes the existence of a fixed-point attractor where the *Apoptosis* variable is activated while all other variables are deactivated. To address the second issue, we design an additional formula φ_3 encoding the dynamic property expressing there should not be any other attractor apart from those that correspond to the programmed cell death phenotype or the experimental data. We thus obtain a new set of dynamic properties $\Omega' = \{\varphi_1, \varphi_2, \varphi_3\}$.

$$\psi_{death} \equiv \mathit{Apoptosis} \wedge (\bigwedge_{\substack{v_i \in Var \\ v_i \neq \mathit{Apoptosis}}} \neg v_i)$$

$$\varphi_2 \equiv \exists y.\, @_y.\ (\psi_{death} \wedge \mathbf{AX}(\psi_{death} \wedge y))$$

$$\varphi_3 \equiv \neg\exists z.\, @_z.\ \neg(\mathbf{AG}\,\mathbf{EF}(\psi_{death} \vee \psi_{data}))$$

Furthermore, we improve the specification of the update logic by substituting the component E of the sketch for a new ("more detailed") partially specified BN E'. We require that when the *Apoptosis* variable is activated, all other variables should be switched off. Therefore, the update function of each network variable v_i (except the *Apoptosis* variable itself) should be $\neg\mathit{Apoptosis} \wedge \boldsymbol{f}_i^{(a)}$, where a is a number of the variable's regulators excluding *Apoptosis*, and $\boldsymbol{f}_i^{(a)}$ represents an uninterpreted Boolean function with these a regulators as its arguments.

When we employ this new refined sketch $S' = (I, E', \Pi, \Omega')$ and run the inference algorithm, only 378 potential consistent networks remain. Individual

Sketch components	S	S'
IG	$3.2e32$	$3.2e32$
IG + PSBN	$3.2e32$	$7.2e16$
IG + PSBN + UFP	$7.8e10$	1296
IG + PSBN + UFP + DP	$9.1e9$	378

Table 3: Numbers of candidates consistent with the two T-LGL model sketches described in the first case study. Each row shows the number of candidates consistent with the particular components of the sketch, starting with just the IG, and considering the whole sketch in the last row.

rows of the third column of Table 3 show the number of candidates consistent with the particular components of the sketch S'. The whole computation for S' only takes less than one second. That is an order of magnitude faster than the computation for S mainly because the searched space of candidates got notably smaller by substituting E for E' (note how the number of candidates consistent with the IG + PSBN components in the Table 3 changes between the two sketches). This demonstrates the importance of the ability to specify the update logic partially using uninterpreted functions. Other similar BN inference approaches usually lack this ability and operate only with either completely unspecified update logic or just with a few specific classes of update functions.

Finally, we examine the similarities between the candidates. By means of automatic analysis, we discover that all consistent candidates agree on the update functions for 13 variables (i.e., for each of these variables, only one consistent update function is possible). Modellers can use this information and only focus on the remaining 5 network components that vary between the candidates. At this stage, the sketch can be refined even more by specifying additional hypotheses, or further experiments may be designed focusing on the varying parts of the network.

4.2 Analysis of the sepal development of A. Thaliana

In our second case study, we consider the model of sepal primordium polarity in the young flower of Arabidopsis thaliana developed in [8]. The model contains 21 variables. Their names are shown in the first column of Table 4. The regulatory interactions in the model were manually created using published data. The authors also defined a set of two expected attractors by analyzing the expression patterns of the most important genes during sepal development. However, after the model was created, the authors were unable to obtain the set of expected attractors. Therefore, they had to add several additional hypothetical regulations. This is an example of a situation where automatic inference tools could be of great importance and help. We show how to employ Boolean network sketches to help with this task.

The case study focusing on this particular model was also conducted by the

Variable	Attractor state 1	Attractor state 2
AGO1	ON	OFF
AGO10	OFF	ON
AGO7	OFF	ON
ANT	ON	ON
ARF4	ON	OFF
AS1	OFF	ON
AS2	OFF	ON
ETT	ON	OFF
FIL	ON	OFF
KAN1	ON	OFF
miR165	ON	OFF
miR390	ON	ON
REV	OFF	ON
TAS3siRNA	OFF	ON
AGO1_miR165	ON	OFF
AGO7_miR390	OFF	ON
AS1_AS2	OFF	ON
AUXINh	ON	ON
CkH	OFF	ON
GTE6	OFF	ON
ITP5	OFF	ON

Table 4: List of variables used in the model of Sepal development of A. Thaliana [8]. The second and third columns show the values of these variables in two expected attractor states [10].

authors of the inference tool Griffin ([10]). We can thus use it as a means to compare some aspects of our approach to theirs (such as the performance). The main difference between the two methods is that Griffin only works with the synchronous semantics of BNs, which considerably simplifies the biological reality. However, note that some dynamic properties, such as fixed-point attractors, are preserved between synchronous and asynchronous semantics, allowing for comparison.

We gradually consider several versions of our sketch, which differ in the dynamic properties. We construct the first version of the sketch to directly compare the performance of our method to that of Griffin, considering the exact same knowledge and data.

We utilise the same signalling network defined in the original article [8] to obtain the influence graph I and generate the set of update function properties Π. The update function properties come from the information on whether the regulations are inhibitions or activations (properties expressing monotonicity) and whether the regulations are hypothetical or not (properties expressing essentiality). To allow for comparison with Griffin, we have to consider PSBN E with completely unspecified update logic since Griffin does not allow partially specifying the update logic.

We define the dynamic properties by utilising the same set of two expected attractor states as in [10]. Values of each variable in these two states are shown in the second and third columns of Table 4. The authors of Griffin considered the expected attractors to be fixed points. We thus automatically construct two HCTL formulae φ_{fixed_1} and φ_{fixed_2}, each encoding the property expressing the existence of the expected fixed-point attractor. To be concise, we use "macros" ψ_{state_1} and ψ_{state_2}, each encoding one of the two states described in Table 4 (for detailed information on encoding binarised observations, see Section 2.1). The set of dynamic properties is then $\Omega = (\varphi_{fixed_1}, \varphi_{fixed_2})$. The whole sketch is $S = (I, E, \Pi, \Omega)$.

$$\varphi_{fixed_1} \equiv \exists x.\, @_x.\ (\psi_{state_1} \wedge \mathbf{AX}(\psi_{state_1} \wedge x))$$
$$\varphi_{fixed_2} \equiv \exists y.\, @_y.\ (\psi_{state_2} \wedge \mathbf{AX}(\psi_{state_2} \wedge y))$$

Griffin computed the set of all consistent networks exhibiting the 2 specified *fixed-point* attractors in 5 hours and 10 minutes. Our method computes the set of all networks consistent with the sketch S in less than half of a second. That means almost 50000x speed up with respect to Griffin. Note that we could not find a working implementation of Griffin, so we use computation times presented in [10], which were obtained on a similar setup (Intel i7 CPU and 16 GB of RAM). Both tools computed the same number of consistent candidates – 439 296.

Since our approach allows us to work with more complex dynamic properties, we modify the sketch. Generally, the expected attractor states do not have to correspond to fixed points but may be a part of more complex attractors. This

leads us to substitute properties φ_{fixed1} and φ_{fixed2} by φ_1 and φ_2, respectively. Moreover, by symbolically analysing the attractors of the candidates consistent with S, we found out that a large part of these candidates exhibits attractors that do not contain any state corresponding to our data. Therefore, we add the formula φ_3 expressing that the candidate network should not exhibit any additional attractors "unrelated" to the data. The modified sketch is then $S' = (I, E, \Pi, \Omega')$, where $\Omega' = (\varphi_1, \varphi_2, \varphi_3)$. Note that all three formulae can again be generated automatically from binarised data.

$$\varphi_1 \equiv \exists x.\, @_x.\ (\psi_{state_1} \wedge \mathbf{AG}\,\mathbf{EF}(\psi_{state_1} \wedge x))$$
$$\varphi_2 \equiv \exists y.\, @_y.\ (\psi_{state_2} \wedge \mathbf{AG}\,\mathbf{EF}(\psi_{state_2} \wedge y))$$
$$\varphi_3 \equiv \neg\exists z.\, @_z.\ \neg(\mathbf{AG}\,\mathbf{EF}(\psi_{state_1} \vee \psi_{state_2})).$$

The utilisation of this new version of dynamic properties causes the number of consistent concretisations to drop to 48 352, so approximately tenfold. Moreover, we can be sure that we did not miss any attractors (since we do not consider only fixed points anymore). The whole computation takes 49 seconds in this case. By means of automatic analysis, we discover that all consistent candidates agree on the update functions for 11 variables (i.e., for each of these 11 variables, only one consistent update function is possible).

4.3 Large biological networks with synthetic attractor data

To evaluate the performance of our method on larger and more complex models, we consider the following scenario. We start with a real-life, fully specified BN F, its corresponding influence graph I, and a set of known properties of update functions (regarding monotonicity or essentiality) encoded into the set Π.

We then compute attractors for F. From the attractors, we derive synthetic steady-state data, emulating experimental observations. For each synthetic observation, we automatically generate a formula encoding the existence of an attractor containing the state corresponding to this observation. We also construct a formula prohibiting any additional attractors that do not contain a state corresponding to any observation (just like in our case studies). The set of considered dynamic properties Ω then contains all these properties.

Finally, we modify F by "relaxing" some of its exact update functions, replacing them with uninterpreted Boolean functions (with the same arity and arguments as in the original model). By doing this, we obtain a partially specified BN E.

We combine the four components to obtain the sketch $S = (I, E, \Pi, \Omega)$. Now we apply the inference algorithm and compute the set of all networks which are consistent with the sketch S. We measure the time needed for the computation. After the process finishes, we also check that the resulting ensemble of networks contains the original network F.

Using this approach, we were able to analyse networks with up to 321 network components. The sketches also admit a large degree of uncertainty –

Model Name	BN Components	Cons. with I, E	Cons. with I, E, Π	Cons. with I, E, Π, Ω	Time
E Protein	35	2^{74}	9.4e5	196	0.5s
NSP4	60	2^{76}	1.2e6	128	0.9s
ETC	84	2^{72}	2.4e8	1.3e6	186.5s
Interferon I	121	2^{103}	5.3e22	6.8e5	58.3s
NSP9	252	2^{356}	7.3e30	6.4e27	39.3s
Macrophage	321	2^{104}	9.9e12	7.8e11	239.8s

Table 5: Overview of several large models and their sketches on which we have successfully run the inference algorithm. For each sketch $S = (I, E, \Pi, \Omega)$, we present how the number of consistent BN candidates changes after more components of the sketch are gradually considered. We omit the number of influence graph candidates. Computation time in seconds is in the last column.

the highest number of admitted candidate networks for a single partially defined BN (component E) was 2^{356}. In Table 5, we present examples of 6 large models we have successfully run the inference algorithm on.

5 Comparison with Related Approaches

In this part, we give a detailed comparison with two most relevant related approaches based on [3] and [13]. The following two sections show that our method generalises the synthesis problems considered by these two publications. Finally, we give a summary of the advantages our method enjoys over these (and other) related approaches.

5.1 Comparison with Chevalier et al.

Now, we compare the theoretical expressiveness of our approach to that of [3, 2]. In particular, we show that every property that the authors consider in [3] can also be encoded into a BN sketch.

First, note that the authors consider an equivalent notion of influence graph and monotonicity properties of regulations. These can be directly translated to our framework using the already presented intuition.

Second, let us observe that our text and [3] agree on the notion of *partial observation* as defined above (i.e. a partial assignment of Boolean values to network variables). As such, we can write that [3] considers a collection of partial observations $o_1, \ldots, o_k$ and a set of associated constraints on these observations. Let us now demonstrate how each of these constraints is expressed in HCTL.

Reachability and non-reachability First, the requirement that o_j is reachable from o_i is similar to the time-series property described in Section 2.1:

$\varphi_{o_i \to o_j} \equiv o_i \wedge \mathbf{EF}\, o_j$. This property holds in all states which match observation o_i and can reach a state matching observation o_j. We can then also invert the property to obtain non-reachability: $\varphi_{o_i \not\to o_j} \equiv o_i \wedge \neg\, \mathbf{EF}\, o_j$. Here, the property holds in states matching o_i that cannot reach any state matching o_j. Finally, note that we can again use $\forall$ or $\exists$ (combined with @) to enhance the formula such that it is valid universally in the whole state space (regardless of the initial state).

Fixed-points and universal fixed-points Expressing that observation o holds in *some* fixed-point of the system is easy in HCTL: $\varphi_{FixedPoint(o)} \equiv {\downarrow}x.\ o \wedge \mathbf{AX}\, x$. This formula is satisfied in states which match o and can only reach themselves (fixed-points). Furthermore, if we want to express that *all* fixed points of the system must match a particular (partial) observation o, we can write that $\varphi_{UniversalFixedPoint(o)} \equiv \forall x.\ @_x\ (\mathbf{AX}\, x) \Rightarrow o$. If there are l different observations that are admissible as the system's fixed-points, we can write $o_1 \vee \ldots \vee o_l$ instead of just o in $\varphi_{FixedPoint}$ and $\varphi_{UniversalFixedPoint}$.

Attractors and universal attractors As already discussed, we can express that a certain partial observation must appear in *some* attractor state using formula $\varphi_{A(o)}$ (see above). Now, let us note that using a similar approach as with universal fixed-points, we can also write that *every* attractor state must match a particular observation: $\varphi_{UniversalAttractor(o)} \equiv \forall x.\ @_x\ (\mathbf{AG}\,\mathbf{EF}\, x) \Rightarrow o$. As was the case for fixed-points, we can swap o for a disjunction of observations when multiple choices are possible.

Reachable universal fixed-points and attractors Finally, we can combine these properties to express reachable universal fixed-points and attractors (we give exact formula for fixed-points, the case of general attractors is similar, swapping $\mathbf{AX}\, x$ for $\mathbf{AG}\,\mathbf{EF}\, x$). In particular, our goal is to express that for all fixed-point states, there is a pair of observations, o_i and o_s, such that the fixed-point matches observation o_s and it is reachable from a state that matches the observation o_i. Formally $\forall x.\ @_x\ ((\mathbf{AX}\, x) \Rightarrow (o_s \wedge \exists y.\ @_y\ \mathbf{EF}\, x))$. Again, if there are multiple possible combinations of o_i and o_s, we can expand the second part of the implication into a disjunction of multiple admissible cases.

5.2 Comparison with Yordanov et al.

Similarly, let us take a look at the work in [13] and compare it to our approach. First, let us note that [13] also considers a compatible notion of influence graph. In particular, their notion of *abstract Boolean network* includes a set of regulations between variables, such that each regulation can have a monotonicity requirement, and can be marked as optional (i.e. the non-optional regulations need to be *essential* in their respective update functions). As we already demonstrated, these types of requirements can be easily expressed in our framework as properties of the network's update functions.

Network inputs and outputs Here, we should also note that [13] explicitly separates network *inputs* and *outputs* from the rest of the variables. However, as far as we understand, this is not critical for the actual specification of the synthesis problem, it just allows to, e.g., specify the observed variable states and assumed input valuation as two separate parts of an overall partial observation (e.g. several observations can share the same input valuation, and so on). As such, this is mostly a syntactical and presentational property of the method. Also, it is worth noting that an input variable as understood in [13] is effectively a logical parameter, which is in our framework better captured using a zero-arity uninterpreted function.

Update function patterns In [13], the method does not actually consider *all* possible Boolean networks that are consistent with the desired influence graph. Instead, the authors define a set of 20 general "patterns" that each update function can take. In our framework, each of these patterns can be expressed as a combination of update function properties and partially defined Boolean expressions.

As an example, consider the first pattern $\mathit{AllActivators}(x) \wedge \mathit{NoRepressors}(x)$ from [13]. Intuitively, a function matches this pattern if it is true exactly when all (essential) activators are active, and all (essential) repressors are inactive. Note that this is not necessarily a single Boolean function, as it is still up to the method to determine which of the optional regulations are essential. However, recall that this is exactly the definition of a *veto* function we describe in Section 1 (including the case where essentiality of all inputs is not mandated, where the *sufficient* properties were used).

Other considered patterns employ similar constructs (i.e. *AllActivators*, *NoActivators*, *AllRepressors* and *NoRepressors*), we can therefore translate these analogously into logical combinations of various *essential* and *sufficient* properties. As our framework allows arbitrary first-order logic formulae, we can simply enumerate all 20 patterns and combine them using a disjunction operator.

Dynamic properties Finally, in terms of dynamic properties, the specification in [13] again relies on the notion of partial observations. Subsequently, the authors can formulate reachability and fixed-point constraints on these partial observations, which we have already described in previous sections.

However, it should be noted that due to the nature of the decision procedure, here the reachability properties are always defined in a bounded sense. That is, the next state must be reachable within some known number of k discrete steps. Meanwhile, our framework does not have this limitation. Nevertheless, a HCTL formula can also express this property by nesting k subsequent **EX** operators instead of a single **EF**. As such, we can artificially introduce this limitation.

5.3 Summary

We have demonstrated that as a specification language, BN sketches are more general than previous methods (in particular, comparing to [3] and [13], which,

to the best of our knowledge, provide the richest specification languages among comparable methods). The expressive power of HCTL allows us to specify rich dynamical assumptions, but more importantly, it allows us to compose and combine these assumptions in a predictable and robust manner that goes beyond the previously employed methods.

Another crucial aspect of BN sketches is our symbolic synthesis procedure which we use to comprehensively explore the whole set of candidate models. Meanwhile, methods like [3] and [13] rely on ASP/SMT solvers and model enumeration, which is inherently limited in the number of explored candidate models. The nature of the symbolic BDD method even allows us to easily share and further refine the set of candidate models (e.g. once additional data is available) without repeating the previous computations.

The solver-based methods enjoy a theoretical performance advantage over our symbolic method (it is sufficient to find a single satisfying BN, instead of *all* BNs). However, as we demonstrate in our case studies, the symbolic approach can still scale to practically sized problems, in which case it provides a more comprehensive and actionable set of results.

References

[1] Nikola Beneš, Luboš Brim, Jakub Kadlecaj, Samuel Pastva, and David Šafránek. AEON: attractor bifurcation analysis of parametrised Boolean networks. In *Computer Aided Verification*, volume 12224 of *Lecture Notes in Computer Science*, pages 569–581. Springer, 2020.

[2] S. Chevalier, C. Froidevaux, L. Pauleve, and A. Zinovyev. Synthesis of Boolean networks from biological dynamical constraints using answer-set programming. In *2019 IEEE 31st International Conference on Tools with Artificial Intelligence (ICTAI)*, pages 34–41. IEEE Computer Society, nov 2019.

[3] Stéphanie Chevalier, Vincent Noël, Laurence Calzone, Andrei Zinovyev, and Loïc Paulevé. Synthesis and simulation of ensembles of Boolean networks for cell fate decision. In *Computational Methods in Systems Biology*, pages 193–209. Springer, 2020.

[4] Gianfranco Ciardo, Gerald Lüttgen, and Radu Siminiceanu. Saturation: An efficient iteration strategy for symbolic state-space generation. In *Tools and Algorithms for the Construction and Analysis of Systems TACAS*, volume 2031 of *Lecture Notes in Computer Science*, pages 328–342. Springer, 2001.

[5] Haleh Ebadi and Konstantin Klemm. Boolean networks with veto functions. *Physical Review E*, 90(2), Aug 2014.

[6] Stephen E. Harris, Bruce K. Sawhill, Andrew Wuensche, and Stuart Kauffman. A model of transcriptional regulatory networks based on biases in the observed regulation rules. *Complex.*, 7(4):23–40, March 2002.

[7] Daniel Kernberger and Martin Lange. Model checking for hybrid branching-time logics. *Journal of Logical and Algebraic Methods in Programming*, 110:100427, 2020.

[8] Camilo La Rota, Jérôme Chopard, Pradeep Das, Sandrine Paindavoine, Frédérique Rozier, Etienne Farcot, Christophe Godin, Jan Traas, and Françoise Monéger. A data-driven integrative model of sepal primordium polarity in arabidopsis. *The Plant Cell*, 23(12):4318–4333, 2011.

[9] Xiang Liu, Yan Wang, Ning Shi, Zhicheng Ji, and Shan He. GAPORE: Boolean network inference using a genetic algorithm with novel polynomial representation and encoding scheme. *Knowledge-Based Systems*, 228:107277, 2021.

[10] Stalin Muñoz, Miguel Carrillo, Eugenio Azpeitia, and David A. Rosenblueth. Griffin: A tool for symbolic inference of synchronous Boolean molecular networks. *Frontiers in Genetics*, 9:39, 2018.

[11] Assieh Saadatpour, Rui-Sheng Wang, Aijun Liao, Xin Liu, Thomas P. Loughran, István Albert, and Réka Albert. Dynamical and structural analysis of a T Cell survival network identifies novel candidate therapeutic targets for large granular lymphocyte leukemia. *PLOS Computational Biology*, 7(11):1–15, 11 2011.

[12] Taisuke Sato and Ryosuke Kojima. Boolean network learning in vector spaces for genome-wide network analysis. In *KR*, pages 560–569, 2021.

[13] Boyan Yordanov, Sara-Jane Dunn, Hillel Kugler, Austin Smith, Graziano Martello, and Stephen Emmott. A method to identify and analyze biological programs through automated reasoning. *NPJ systems biology and applications*, 2(1):1–16, 2016.

[14] Ranran Zhang, Mithun Shah, Juncheng Yang, Susan Nyland, Xin Liu, Jong Yun, Réka Albert, and Thomas Loughran. Network model of survival signaling in LGL leukemia. *Proceedings of the National Academy of Sciences of the United States of America*, 105:16308–13, 2008.
